# Gender-Based Differences in Anthropometry and Cord Blood Insulin Levels in Term Neonates

**Published:** 2014-01-13

**Authors:** Subarna Mitra, Prasanta K Nayak, Jayaprakash Sahoo, Sujata Misra, Sadishkumar Kamalanathan

**Affiliations:** 1 Department of Obstetrics & Gynaecology, SCB Medical College, Odisha; 2 Department of Obstetrics & Gynaecology, All India Institute of Medical Sciences, Raipur, Chhattisgarh; 3 Department of Endocrinology & Metabolism, JIPMER, Puducherry, India

**Keywords:** Anthropometry, Birth Weight, Cord Blood Insulin, Large for Gestational Age, Appropriate for gestational age

Gender has been unmasked as a key determinant of body adiposity and endocrine homeostasis in the human fetus. Increasing evidence suggests that girls are more insulin resistant than boys at all ages from birth to adolescence^[^^1^^-^^3^^]^. Furthermore, type 2 diabetes in children is commoner in girls than in boys^[^^4^^]^. These gender based differences seen early in life could reflect differences in intrinsic insulin resistance or postnatal behavior.

 We had conducted a study, published elsewhere, to determine the effects of maternal anthropometry and metabolic parameters on fetal growth[5]. In a post-hoc analysis, we aimed to determine whether any gender based difference in anthropometry or insulin levels exists in Indian children. ^[^^5^^]^. Out of the 50 neonates, 26 were males (18 AGA and 8 LGA) and 24 were females (22 AGA and 2 LGA). The neonatal and maternal parameters are depicted in Table 1. The mean cord blood insulin levels were 15.15±15.93 mIU/L in males and 11.77±10.95 mIU/L in females, respectively. There was no statistically significant difference in any anthropometric or metabolic parameter between the two groups. According to current literature, the average weight, length, and HC of girls are lower than that of boys, but girls are more adipose with higher circulating levels of insulin at term^[^^6^^-^^8^^]^. But we did not find any statistically significant difference in anthropometry between male and female babies, in accordance with few studies^[^^8^^,^^9^^]^. Also, our study did not demonstrate any gender specificity in cord blood insulin levels, similar to several reports^[^^9^^,^^10^^]^.

 There can be several explanations for lack of sex differences in anthropometry and umbilical cord insulin concentrations. Maternal anthropometry, glycemic status and insulin levels are important determinants of fetal growth. In our earlier report, we had concluded that maternal BMI is the most important predictor of birth weight and that maternal serum and cord blood insulin levels are correlated with each other^[^^5^^]^. Absence of gender specificity in maternal anthropometry and serum insulin levels (Table 1) possibly accounted for absence of sexual dimorphism in neonatal anthropometry and cord

**Table 1:** Maternal and neonatal parameters

**Parameter**	**Male neonates (26)**	**Female neonates (24)**	***P*** **. value**
**Maternal Age (yr)**	25.27 (3.78)	25.4 (3.36)	0.8
**Gestational Age (wk)**	39.44 (1.22)	39.16 (1.15)	0.07
**Parity (one)**	24 (92%)	17 (71%)	0.07
**Maternal Weight (kg)**	56.77 (7.53)	56.56 (7.19)	0.8
**Maternal Height (m)**	1.52 (0.03)	1.52 (0.04)	0.8
**Maternal BMI (kg / m** ^2^ **)**	24.47 (3.25)	24.20 (2.92)	0.4
**Maternal RBS (mg/dL)**	90.96 (37.33)	88.03 (30.06)	0.5
**Maternal serum insulin (mIU/L)**	20.64 (16.27)	16.50 (13.86)	0.2
**Neonatal Weight (kg)**	3.08 (0.54)	2.92 (0.38)	0.1
**Neonatal Length (m)**	0.48 (0.02)	0.47 (0.01)	0.1
**Ponderal index (kg/m** ^3^ **)**	27.72 (3.23)	27.57 (3.82)	0.4
**Head circumference (cm)**	34.09 (1.73)	33.23 (1.57)	0.07
**Abdominal circumference (cm)**	30.33 (2.64)	30.59 (2.05)	0.3
**Chest circumference (cm)**	32.77 (2.11)	32.81 (1.26)	0.5
**Cord blood insulin(mIU/L)**	15.15 (15.93)	11.77 (10.95)	0.2

BMI: Body mass index; RBS: Random blood glucose

blood insulin levels in our study. Secondly, cord blood insulin concentrations decrease if collected in heparin and stored at room temperature^[^^1^^]^. But in our study, the samples were collected in EDTA and immediately refrigerated. The third explanation could be the pulsatile release and shorter half-life of insulin coupled with its possible

fluctuations during delivery^[^^1^^]^.

 To conclude, neither neonatal anthropometry nor cord insulin levels show sexual dimorphism at birth among Indian children. Being an observational cross-sectional study with limited sample size, our results need validation from larger studies. 
